# The Guild of St. Thomas

**Published:** 1920-11-06

**Authors:** 


### The Guild of St. Thomas.

At the invitation of the Dowager Countess of Derby, the members of this most useful body assembled recently at the Treasurer's House, St. Thomas's Hospital, to hold the annual business meeting. There was a large gathering.

The chair was occupied by Mrs. W. H. Battle, and among those who supported her were the Hon. Sir Arthur Stanley, Lady Wallace, Lady Riddell, Mrs. Kendal, Lady Gathorne Hardy, Lady Ballance, Mr. Roberts (secretary of the hospital), and Miss Lloyd Still (matron of St. Thomas's).

Mrs. Battle, in opening the proceedings, referred sympathetically to the demise of the Dowager Duchess of Bedford, who had taken a very kindly interest in the Guild's activities, and more than once presided at their meetings. Her encouragement and help would remain a fragrant memory.

Mrs. Edred Corner, the honorary secretary, read the report of the year's work, pointing out the restriction of output caused by the prevailing high prices of materials of all kinds. A special appeal had not rendered what was hoped. Still, the members had this year made 2,055 garments and handed them over to the hospital. During the year twelve working parties had been held, and thanks were offered to the various hostesses at whose homes members met for the work. Last year twenty-nine new members were reported, and so far this year the accession had been thirteen. An appeal was made to a wider circle for help in material and money to enable the work to be extended; there was great need of garments among those who were discharged from the hospital or were convalescing. A pathetic instance of this was related of one of the cleaning women, who, on being questioned, admitted she had no underclothing, and that one pair of shoes was being shared by her daughter, her husband having been for long out of work. In the present time of high wages there was just a danger of forgetting that such cases could still exist. Annually some 700 garments were given away, in addition to those supplied to occupants of the various wards.

A proposal was then duly carried that the minimum subscription for future members be 5s. instead of 2s. 6d.

After an address from Mrs. Kendal, Miss Lloyd Still tendered to the Guild, on her own behalf and that of the sisters, warm thanks for all that it had done for the necessitous poor patients; it was a great thing to go to the cupboard and, however great the drain had been, find it constantly replenished.

Mrs. Kendal sprang an agreeable surprise on the meeting just as it was dispersing by stating she would give £50 towards making up the year's deficit.

